# Nasopharyngeal Carriage and Transmission of *Streptococcus pneumoniae* in American Indian Households after a Decade of Pneumococcal Conjugate Vaccine Use

**DOI:** 10.1371/journal.pone.0079578

**Published:** 2014-01-17

**Authors:** Jonathan F. Mosser, Lindsay R. Grant, Eugene V. Millar, Robert C. Weatherholtz, Delois M. Jackson, Bernard Beall, Mariddie J. Craig, Raymond Reid, Mathuram Santosham, Katherine L. O'Brien

**Affiliations:** 1 Center for American Indian Health, Johns Hopkins Bloomberg School of Public Health, Baltimore, Maryland, United States of America; 2 Infectious Disease Clinical Research Program, Uniformed Services University of the Health Sciences, Bethesda, Maryland, United States of America; 3 Division of Bacterial Diseases, National Center for Immunization and Respiratory Diseases, Centers for Disease Control and Prevention, Atlanta, Georgia, United States of America; 4 White Mountain Apache Tribe, Whiteriver, Arizona, United States of America; Columbia University, United States of America

## Abstract

**Background:**

Young children played a major role in pneumococcal nasopharyngeal carriage, acquisition, and transmission in the era before pneumococcal conjugate vaccine (PCV) use. Few studies document pneumococcal household dynamics in the routine-PCV7 era.

**Methods:**

We investigated age-specific acquisition, household introduction, carriage clearance, and intra-household transmission in a prospective, longitudinal, observational cohort study of pneumococcal nasopharyngeal carriage in 300 American Indian households comprising 1,072 participants between March 2006 and March 2008.

**Results:**

Pneumococcal acquisition rates were 2–6 times higher in children than adults. More household introductions of new pneumococcal strains were attributable to children <9 years than adults ≥17 years (p<0.001), and older children (2–8 years) than younger children (<2 years) (p<0.008). Compared to children <2 years, carriage clearance was more rapid in older children (2–4 years, HR_clearance_ 1.53 [95% CI: 1.22, 1.91]; 5–8 years, HR_clearance_ 1.71 [1.36, 2.15]) and adults (HR_clearance_ 1.75 [1.16, 2.64]). Exposure to serotype-specific carriage in older children (2–8 years) most consistently increased the odds of subsequently acquiring that serotype for other household members.

**Conclusions:**

In this community with a high burden of pneumococcal colonization and disease and routine PCV7 use, children (particularly older children 2–8 years) drive intra-household pneumococcal transmission: first, by acquiring, introducing, and harboring pneumococcus within the household, and then by transmitting acquired serotypes more efficiently than household members of other ages.

## Introduction

Pneumococcal conjugate vaccines are being introduced into routine childhood immunization schedules around the world, including in low-resource countries. Routine infant vaccination with heptavalent pneumococcal conjugate vaccine (PCV7; Prevnar [Pfizer]) has substantially reduced pneumococcal disease in the United States [1]–[4] and Europe [5]. Still, in 2008, approximately 514,000 children under 5 years of age died from pneumococcal disease; over 90% of these deaths occurred in developing countries where PCV had not yet been introduced [6].

Nasopharyngeal (NP) colonization with *Streptococcus pneumoniae* plays a major epidemiologic role both as a disease precursor [7], [8] and the primary mode of horizontal transmission between individuals [9], with the household as a primary mixing pool [10]. Pre-PCV longitudinal NP colonization studies suggest that children introduce new serotypes into the household more frequently, acquire household serotypes more often, and carry serotypes longer than adults [10]–[14]. Serotype-specific NP carriage is highly temporally clustered within households [15], [16], and household exposure increases serotype-specific acquisition in other household members eight-fold [17]. Serotypes also differ intrinsically by carriage duration, intra-household transmissibility, and resistance to colonization by competing serotypes [11], [14].

Longitudinal studies of intra-household pneumococcal NP transmission have been conducted prior to PCV7 introduction [15], [16], [18] or in countries where PCV7 is not widely available [11], [17] but not in the setting of PCV use. PCV reduces vaccine-serotype (VT) carriage in both vaccinated and unvaccinated children [19], [20] and adults [21], but high carriage prevalence or suboptimal vaccine coverage may attenuate these indirect effects [22]. Widespread PCV use may substantially alter pneumococcal epidemiology within households, exerting differential effects by vaccination status and population under evaluation.

This longitudinal study aimed to characterize pneumococcal NP acquisition, carriage duration, and transmission in American Indian households during the routine PCV7 era. PCV7 was first introduced to these communities in 1997 during a group-randomized efficacy trial [23], then included in the routine infant immunization schedule immediately afterwards in 2000 [24]. PCV7 has virtually eliminated VT carriage [25] and dramatically reduced VT IPD (invasive pneumococcal disease), although residual IPD rates remain four-fold those of the general US population [24]. Since nearly all circulating pneumococci in these communities are now non-vaccine serotypes (NVT), we did not expect individual child vaccination status to influence household pneumococcal dynamics in this routine-PCV7 setting. We hypothesized that children would serve as the major route of pneumococcal serotype introduction into the household and the primary source of intra-household transmission, just as in the pre-PCV7 era. Furthermore, characterization of household transmission in this high-burden population may predict perturbations in pneumococcal ecology likely to occur in other high-burden populations worldwide that are introducing PCV.

## Methods

### Ethics Statement

The study was approved by the Navajo Nation, the White Mountain Apache tribe and the institutional review boards of the Johns Hopkins Bloomberg School of Public Health, the Navajo Nation, and the Phoenix Area Indian Health Service. Written informed consent was obtained from all adult participants and parents or guardians of enrolled children.

### Longitudinal Nasopharyngeal Colonization Study

The study protocol has been previously described [25]. Briefly, American Indian families with at least one child <9 years and living on or near the Navajo or White Mountain Apache reservations in the southwestern United States were enrolled between March 2006 and March 2008, then visited monthly for six months (seven total visits). Household carriage risk factors and family composition were ascertained at enrollment; individual carriage risk factors were collected at each visit. Pneumococcal immunization history and records of antibiotic use, hospitalizations, and (for children <2 years) outpatient illnesses during the study period were collected via medical chart review. Monthly NP specimen collection, pneumococcal isolation, and serotyping were conducted as previously described, allowing identification of multiple serotypes per swab when multiple morphologies were identified on a plate [25]–[27]. Among isolates originally classified as 6A, Quellung reaction [28], [29] or polymerase chain reaction [27] was used to identify serotype 6C isolates. Three isolates originally classified as 6A were not available for retesting and were assumed to be 6C based on the testing results of other isolates.

### Definitions and Statistical Methods

Daycare attendance was defined as ≥4 hours per week outside of the home with ≥2 members of another household. PCV7 vaccination was defined as ≥3 doses. 23-valent pneumococcal polysaccharide (PS23; Pneumovax [Merck]) vaccination was defined as any previous receipt of PS23.

A NP *specimen* refers to a single sample from an individual; an *isolate* refers to a pneumococcal strain, specified by its serotype identified from a swab. A *carriage episode* refers to the isolation of a serotype from an individual along with as any subsequent isolation of that same serotype at consecutive visits. Carriage episodes were *incident* if preceded by ≥1 swab negative for that serotype and *prevalent* otherwise. Serotype *acquisition* occurred when an isolated serotype was immediately preceded by ≥1 swab negative for that serotype. Acquisitions were *individual new acquisitions* if the individual had never previously carried the serotype in the study, and *individual reacquisitions* otherwise. Acquisition of a serotype never previously observed among participating household members was a *household introduction*. Household introductions were *unique* if a single household member acquired the new serotype and *concurrent* if simultaneously acquired by >1 member. In order to investigate serotype-specific differences in household introduction, unadjusted categorical binomial regression was used to analyze the serotype-specific proportion of concurrent versus unique household introductions for the ten most-commonly-introduced serotypes (with all other serotypes as the reference category).

### Statistical Methods

To analyze carriage duration, incident carriage episodes were aligned for survival analysis on a time metric of “days from first observed carriage”. Clearance was defined by two consecutive swabs negative for a carried serotype and assumed to occur at the midpoint between the last swab positive and the first swab negative for that serotype. Carriage episodes concluding with a missed swab or the last study visit were right-censored. Unadjusted median time to clearance was calculated using survival analysis. Cox regression with cluster robust standard errors was used to analyze the effect of age on clearance of a carried serotype, using only the first incident observation per individual and adjusting for time-varying (breastfeeding, tobacco or antibiotic use, daycare attendance, and visit month) and time-fixed (household running water, tobacco smoke, or indoor stove; vaccination status; number of household members [total and <6 years]; and persons per sleeping room) covariates. To examine whether clearance rate was affected by serotype in children, the above Cox regression was repeated for children <9 years, limiting to the first acquisition of one of the five most commonly carried serotypes. Serotype was included in the regression as a categorical variable and its inclusion tested for significance using a Wald test. Serotype values were carried forward for missed visits only if isolation of that same serotype both preceded and followed the missed visit.

Individual acquisition and household introduction rates were compared using Poisson regression with cluster robust standard errors. Exposure was defined as [number of swab specimens obtained–1] for all Poisson models, since acquisition could not occur at first visit by definition. All analyses were conducted in Stata 12 [30].

Multilevel analysis was used to investigate age-specific intra-household transmission patterns for the four serotypes with the greatest number of putative transmission events (6C, 19A, 22F, and 35B). For each serotype, separate three-level (visit, individual, household) mixed logistic regression models for children and adults were fitted [31], [32], specifying random intercepts for household and individual with an independent correlation structure. Visit-level adjustment for seasonality was performed using a truncated Fourier series [33] (including the first two pairs of terms as independent variables).

## Results

### Household and individual characteristics

A total of 1072 individuals in 300 households were enrolled in the study. *Table 1* summarizes household and individual characteristics. Vaccination coverage was high (83.5%) among children <9 years, while daycare attendance was rare (4.7%). Of 299 households self-reporting household size, 69 (23%) had all persons ≥6 years enrolled, 77 (26%) were missing one person ≥6 years, 55 (18%) were missing two, and 98 (33%) were missing three or more. Enrollment was more complete among younger children: in 210 households (70%), all children <6 years were enrolled, in 74 (25%) one child <6 years was not enrolled and only 15 (5%) households had two or more children <6 years not enrolled.

**Table 1:** Characteristics of individuals and households enrolled in the study.

Household characteristics (n = 300)	Median	IQR
# of members (by interview)	6	[??5, 7]
# of reported members not enrolled in study	2	[??1, 3]
# of members under age 6 (by interview)	2	[??1, 3]
# of reported members under age 6	0	[??0, 1]
not enrolled in study		
# rooms in house	6	[??4, 7]
# sleeping rooms in house	3	[??2, 3]

Household risk factors	Proportion of households	%
Running water	250/299	83.6%
Toilet	245/299	81.9%
Smoker in house	78/297	26.3%
Wood-burning stove	184/298	61.7%

Individual characteristics: children (#, % of enrollees in age category)	<2 years		2–4 years		5–8 years^b^		All children <9 years	
	#	%	#	%	#	%	#	%
Male	118	50.9%	114	51.4%	89	52.7%	321	51.5%
Breastfed during study	72	31.0%	19	8.2%	4	2.4%	95	15.2%
Daycare during study^a^	10	4.3%	16	6.9%	3	1.8%	29	4.7%
Otitis media during study	69	29.7%	26	11.2%	12	7.7%	108	17.3%
Antibiotics during study	75	32.3%	38	16.4%	19	11.2%	132	21.2%
PCV7 (3 doses)	192	82.8%	206	92.8%	121	72.0%	520	83.5%
Total in age category (% of all participants)	232	21.6%	222	20.7%	169	15.6%	623	58.1%

Individual characteristics: adults (#, % of enrollees in age category)	17–39 years^c^		40–64 years		≥65 years		All adults ≥17 years	
	#	%	#	%	#	%	#	%
Male	84	22.2%	13	21.1%	2	22.2%	99	22.0%
Antibiotics during study	49	12.9%	10	16.4%	1	9.1%	60	13.4%
Smoked during study	56	14.8%	5	1.3%	0	0.0%	61	13.6%
Chewed tobacco during study	24	6.4%	3	0.8%	1	0.3%	28	7.4%
PS23 (any previous administration)^d^	18	4.8%	17	27.9%	8	72.7%	43	9.6%
Total in age category (% of all participants)	379	35.3%	61	5.7%	9	0.8%	449	42.1%

^a^ Daycare attendance was defined as ≥4 hours per week outside the home with 2 or more children from other households.

^b^ Includes one child 10 years of age.

^c^ Includes two parents <17 years.

^d^ An additional 4 children <9 years of age reported receipt of PS23.

### NP specimens and carriage

Of the 7504 NP specimens anticipated from the 1072 participants, 6541 (87%) were successfully collected. Most (61%) participants provided all seven swabs, and 88% provided ≥5. A comprehensive description of prevalence and serotype distribution has been published [25]. Carriage prevalence was high: 35.8% overall and 55.5% in children <5 years.

The 2412 pneumococcal isolates represented 1655 distinct carriage episodes; 400 (24.2%) were prevalent and 1255 (75.8%) incident. Per-swab carriage probabilities are summarized in *Table 2*. Among the 945 individuals with ≥5 swabs, 72% carried pneumococcus at least once during the study. The median number of unique serotypes carried by those with ≥5 swabs was 1 (IQR 0, 2), with carriage of ≥4 unique serotypes observed in 6.4%. Of those with ≥5 swabs, 92.5% of children <9 years carried pneumococcus at least once, compared to 41.9% of adults ≥17 years (p<0.0001). On average, 3.18 different serotypes were observed in a household over the study (SD 1.86), with carriage observed at least once in 94% of households. Circulation of >1 serotype over the course of the study was observed in 74% of households. Compared to households with carriage observed at least once, households without pneumococcal isolation had fewer self-reported members (median: 4.5 versus 6.0, Wilcoxon rank-sum p = 0.0076) and fewer children <6 years (median: 1 versus 2, Wilcoxon rank-sum p = 0.0009). Of 1922 household-visits with ≥1 NP swab obtained from the household, the number of serotypes detected in the household was zero at 32.1%, one at 45.4%, and two or more at 22.5% of household visits.

**Table 2:** Summary of pneumococcal acquisition events by age category.

	Age category													
	<2 years		2–4 years		5–8 years		17–39 years		40–64 years		≥65 years		All ages	
**Swabs obtained** *(#)*	1447		1379		1035		2233		376		58		6541	
**Total # isolates** a *(#, per-swab probability)*	835	0.577	787	0.571	502	0.486	249	0.111	36	0.096	8	0.138	2417	0.37
**Individual acquisitions** a *(#,per-swab probability)*	369	0.301	426	0.367	285	0.326	151	0.078	21	0.065	5	0.098	1257	0.227
New acquisitions^b^ *(#, % individual acquisitions)*	282	76.4%	357	83.8%	229	80.4%	133	88.1%	21	100.0%	4	80.0%	1026	81.6%
Reacquisitions^b^ *(#, % individual acquisitions)*	87	23.6%	69	16.2%	56	19.6%	18	11.9%	0	0.0%	1	20.0%	231	18.4%
**Household introductions** a ^,^ c *(#,per-swab probability)*	175	0.143	249	0.215	173	0.198	70	0.036	9	0.028	3	0.059	679	0.123
Unique HH introduction *(#, % of HH introductions)*	102	58.3%	165	66.3%	125	72.3%	36	51.4%	5	55.6%	1	33.3%	434	63.9%
Concurrent HH introduction *(#, % of HH introductions)*	73	41.7%	84	33.7%	48	27.7%	34	48.6%	4	44.4%	2	66.7%	245	36.1%

^a^ Including 73 swabs for which two serotypes were identified.

^b^ New acquisitions defined as acquisition of serotype not previously carried; reacquisition defined as acquisition of a previously-carried serotype.

^c^ Household introductions classified as “unique” if the participant was the only one in the family to acquire the new serotype; “concurrent” if more than one individual acquired the serotype simultaneously.

Concurrent serotype-specific carriage within households was also common: for 41.7% of isolates, ≥1 other household member simultaneously carried the same serotype. Among children <9 years, there was no difference between females and males in the proportion of isolates concurrently carried by other household members (42.1% of isolates from female children versus 39.4% from males, p = 0.204). Among adults ≥17 years, however, concurrent household carriage was significantly more common among adult females than males (53.1% versus 33.3% of isolates, p = 0.006).

### Duration of carriage

Most carriage episodes (70.9%, 1170/1651) were observed at a single visit. Four individuals <5 years carried the same serotype at all seven study visits (two 6C and one each 19A and 33F). Compared to children <2 years, older children and adults cleared carried serotypes at a greater rate (2–4 year-olds, adjusted hazard ratio [HR] of clearance 1.53 [95% CI: 1.22, 1.91]; 5–8 year-olds, HR 1.71 [1.36, 2.15]; adults ≥17 years, HR 1.75 [1.16, 2.64]). Of the five most-commonly carried serotypes, serotype did not significantly affect the hazard of clearance among children <9 years (p = 0.13) or adults ≥17 years (p = 0.37).

### Individual acquisitions

**Figure 1:**
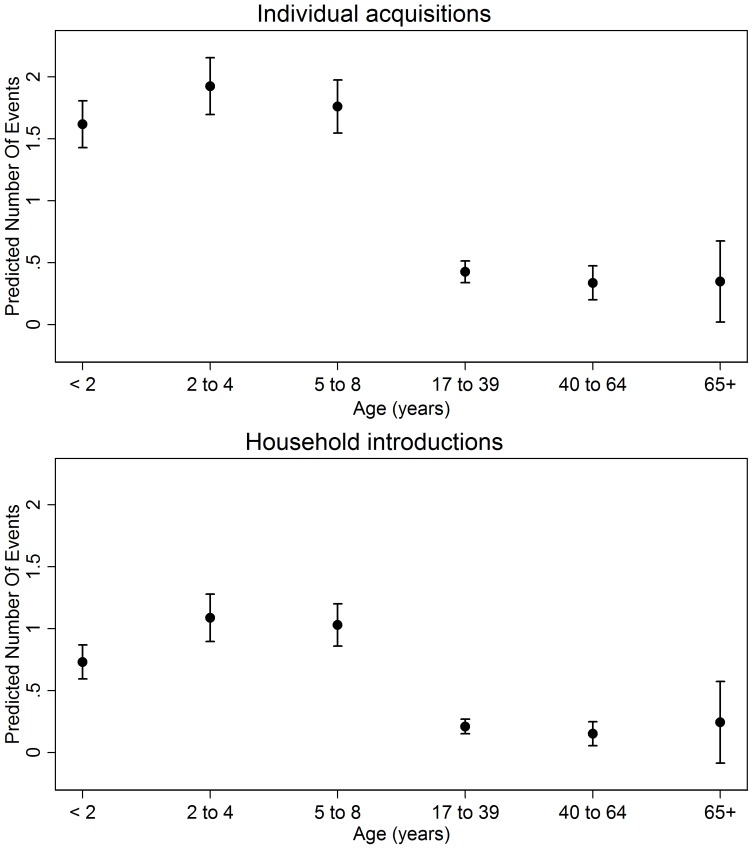
Adjusted number of pneumococcal acquisition events, by age category. Each point represents the mean number of individual acquisitions or household introduction for an individual in a given age category (with 95% confidence interval) over the course of the study, adjusted by all other covariates in the Poisson models set to their means.

*Table 2* summarizes per-swab acquisition probabilities by age, while *Figure 1* shows the adjusted mean number of events by age. At least one individual acquisition was observed in 66.8% (632/945) of individuals who contributed ≥5 swabs and 89.3% (268/300) of all households. After adjusting for individual and household characteristics, the rate of individual acquisition was 3–4 times higher in children <9 years compared to adults 17–39 years (*Table 3*). No significant differences were seen between adult age groups. Month of study enrollment was significantly associated with individual acquisition (p<0.001), with adjusted acquisition rates twice as high in those who enrolled in September compared to February (*Figure S1*). Of other risk factors, only the association between rate of acquisition and reported number of children <6 years in the household reached significance (incidence rate ratio [IRR] 1.09 for each additional child <6 years, 95% CI: 1.02, 1.16).

**Table 3:** Risk factors for pneumococcal acquisition (Poisson regression).

		Individual acquisition			Household introduction		
Individual characteristics		IRR	[95% CI]	p-value	IRR	[95% CI]	p-value
Age (years)	<2	**3.79**	**[2.92,4.93]**	**<0.001**	**3.47**	**[2.36,5.11]**	**<0.001**
	2–4	**4.51**	**[3.47,5.88]**	**<0.001**	**5.16**	**[3.54,7.53]**	**<0.001**
	5–8	**4.13**	**[3.20,5.34]**	**<0.001**	**4.89**	**[3.42,6.99]**	**<0.001**
	17–40	REF			REF		
	40–65	0.79	[0.51,1.22]	0.289	0.73	[0.37,1.41]	0.343
	≥65	0.82	[0.31,2.12]	0.676	1.17	[0.31,4.34]	0.819
Sex (male)		1.01	[0.91,1.11]	0.889	1.07	[0.92,1.25]	0.368
During study^a^:	Breastfed	1.07	[0.92,1.23]	0.392	1.01	[0.81,1.27]	0.926
	Attended daycare	0.85	[0.70,1.03]	0.093	0.96	[0.75,1.23]	0.728
	Smoked tobacco	0.78	[0.50,1.20]	0.252	0.78	[0.42,1.44]	0.43
	Chewed tobacco	1.45	[0.84,2.49]	0.183	1.01	[0.39,2.66]	0.977
Vaccination status^b^	PCV7 (>3 doses)	0.94	[0.79,1.12]	0.493	1.1	[0.85,1.42]	0.452
	PS23 (any)	1.12	[0.70,1.78]	0.636	1.17	[0.53,2.57]	0.702
Month of Study Entry^c^				**<0.001**			**<0.001**
Household characteristics	# children <6	1.09	[1.02,1.16]	0.009	0.93	[0.85,1.00]	0.063
	All other household covariates^d^			NS			NS

^a^ Reported at least once during the study. Daycare defined as ≥4 hours/week outside of home with ≥2 members of other families.

^b^ From chart review; all other covariates ascertained via interview.

^c^ All models adjusted for month of study entry as a categorical variable.

^d^ Estimates additionally adjusted for # persons in household, crowding (number of persons per sleeping room), presence of smoker, presence of wood burning stove, and presence of running water in house. None of the other variables examined had a significant association with either individual or household carriage.

### Household introductions

Five hundred fifty-one (551) household introductions were observed; 78.8% (434/551) were unique and 21.2% (117/551) were concurrent. Of the concurrent introductions, two household members simultaneously acquired the serotype on 105 occasions, three on 11 occasions, and four on one occasion. Of individuals with ≥5 swabs, 48.3% (457/945) had at least one household introduction. For the ten most commonly introduced serotypes in this study, there was no difference in the proportion of concurrent versus unique household introductions by serotype – except for serotype 15A, which was more commonly concurrently introduced (44.0% of introductions, p = 0.005) than other serotypes.

In multivariable Poisson analysis, the rate of new household introduction was 3–5 times higher in children <9 years compared to adults 17–39 years and varied significantly with month of study entry (*Table 3* *; Figure S1*). No other risk factor reached statistical significance.

In a secondary analysis allowing the effect of gender to differ between children <9 years and adults ≥17 years, adult males had a higher rate of household introduction than adult females (IRR 1.72, 95% CI: 1.03, 2.87), while the impact of gender among children was non-significant (IRR 1.02 for males versus females, 95% CI: 0.87, 1.19).

#### Intra-household transmission

Considering serotypes 6C, 19A, 35B, and 22F, exposure to carriage in a child 2–8 years was most consistently associated with a significant increase in the odds of other household members acquiring that serotype at the next visit (*Table 4*). This association was observed for serotypes 19A and 22F considering children <9 years as potential recipients and for 19A, 22F and 35B considering adults ≥17 years. Exposure to carriage in children 2–8 years appeared to have a greater effect on acquisition for adults than children, although wide, overlapping confidence intervals and differences in model structure precluded direct comparison. The only other pair to reach statistical significance was exposure of adults ≥17 years to serotype 6C carriage in children <2 years (OR 19.55, 95% CI: 4.36, 87.69).

**Table 4:** Intra-household pneumococcal transmission (multilevel logistic regression.

		OR for acquisition [95% CI] with p-value, by serotype^b^											
		6C			19A			35B			22F		
Recipient	Exposure^a^	OR	[95% CI]	p-value	OR	[95% CI]	p-value	OR	[95% CI]	p-value	OR	[95% CI]	p-value
Child (<9 y)	No HH carriage	REF			REF			REF			REF		
	Child (<2 y)	1.08	[0.37,3.13]	0.89	1.04	[0.34,3.15]	0.95	1.98	[0.49,8.06]	0.34	2.43	[0.71,8.25]	0.156
	Child (2–8 y)	2.30	[0.90,5.86]	0.08	**3.80**	**[1.66,8.70]**	**0.002**	3.00	[0.93,9.65]	0.065	**6.93**	**[2.16,22.19]**	**0.001**
	Adult (≥17 y)	0.29	[0.03,2.82]	0.28	0.88	[0.13,5.86]	0.89	2.53	[0.12,51.98]	0.55	1.48	[0.24,9.11]	0.673
Adult (≥17 y)	No HH carriage	REF			REF			REF			REF		
	Child (<2 y)	**19.55**	**[4.36,87.69]**	**<0.001**	5.63	[0.86,36.63]	0.071	1.72	[0.17,17.93]	0.65	**5.59**	**[1.05,29.87]**	**0.044**
	Child (2–8 y)	3.69	[0.83,16.33]	0.085	**10.36**	**[1.80,59.45]**	**0.009**	**43.68**	**[7.41,257.6]**	**<0.001**	**20.99**	**[4.90,89.95]**	**<0.001**

^a^ Exposure defined as carriage of the target serotype in at least one household member in the specified age range at the previous visit.

^b^ Both child recipient and adult recipient models adjusted for seasonality (using the first two pairs of a truncated Fourier series), recipient characteristics (age, sex) and household characteristics (number of children <6 y, wood- or coal-burning indoor stove, and running water). Child recipient models also adjusted for vaccination status (defined as at least 3 doses of PCV7), and breastfeeding status (since last visit). Low PS23 coverage in adult age groups and the rarity of serotype-specific adult-to-adult exposure precluded the addition of PS23 vaccination status and exposure to carriage in adults ≥17 years to the adult model. Visits in which adults 17 years were exposed to carriage in another adult ≥17 years at the previous visit were excluded from the adult model.

## Discussion

This study is the largest longitudinal household analysis of pneumococcal carriage and transmission to date and the first conducted in the setting of routine PCV use. Concordant with pre-PCV7 household studies, our findings support a major role for children in pneumococcal acquisition and transmission in the household, but we have now shown that this holds true even for settings of PCV use and high pneumococcal transmission.

Numerous studies indicate that duration of carriage decreases with increasing age [7], [15], [11], [13], [34]. Our findings suggest that trend is unchanged in the presence of routine PCV use. Overall carriage duration was generally similar to that observed in this population prior to the PCV7 introduction [26], despite changes in serotype distribution. These results suggest that older children continue to serve as important reservoirs of pneumococcal carriage within households when PCV is routinely used. Unlike other household studies [11], [34], we did not detect a significant difference in carriage duration by serotype. This observation may be an artifact of our monthly sampling frame, which is biased toward detection of serotypes with longer durations of carriage and limited our ability to resolve finer distinctions in carriage duration.

Children have previously been observed to be the major route of introduction of pneumococcus into the household, both in developing [11] and developed [14] settings. Our findings indicate that this role continues after routine PCV use, across all of the most commonly carried serotypes in this study. Moreover, older children appear to play a larger role in household introductions than children <2 years, likely due to increased exposure in the community and school. In contrast, children <2 years were the primary source of household introduction in a pre-PCV cohort from the United Kingdom [35], perhaps reflecting differences in serotype distribution or age-specific exposure and acquisition patterns between study populations. Other household introduction analyses have not compared children by age subgroup [11], [14].

Among adults in this community, men may introduce serotypes into the household at a higher rate than women; conversely, women may be more involved in intra-household transmission (given their higher rates of concurrent carriage). These findings may reflect higher rates of community exposure in men and effective household contact in women or a bias in the subset of men who participated in the study compared with women.

By isolating the effect of age on transmission – independent of age-specific carriage prevalence – multilevel analysis further confirmed the importance of older children in pneumococcal household dynamics, especially as transmitters to adults. Interestingly, for serotype 6C, exposure to carriage in children <2 years was significantly associated with adult acquisition, while exposure to carriage in older children was not. This observation may be due to imprecision alone or a true difference in serotype-specific transmissibility patterns. In a recent pre-PCV7 Kenyan study, mothers and siblings of all ages transmitted to neonates with similar efficiency [36]. While our study did not specifically examine this pairing, exposure to carriage in children 2–8 years here tended to be more strongly associated with acquisition in children <9 years than carriage in other age groups.

Our study has several limitations. Risk factor data was collected via interview, potentially introducing reporting bias. Most households had members who declined to participate, especially older adults and especially men. Our analyses adjusted for reported household composition and accounted for the number of swabs obtained by age category. Still, we cannot exclude the possibility that observed acquisitions may have been transmitted from missing household members. Our inferences assume that consecutively carried or transmitted serotypes represented the same pneumococcal strain. Multi-locus sequence typing or whole genome sequencing may provide stronger strain-specific inferences than serotyping alone [11]. Although our methods were capable of detecting multiple serotypes per swab when more than one morphology was present, our methods were unable to evaluate the impact of strain-strain competition within the nasopharynx. Our analysis does not distinguish between immune-mediated clearance and displacement of one serotype by another, two biologically distinct processes which are likely to have differential impacts on our observed clearance rate by age group and risk factor under study. Even in the absence of complete displacement, if a serotype were reduced below our threshold of detection by proliferation of a second, co-colonizing serotype, our analysis would register clearance of the former and an acquisition of the latter, rather than continued co-colonization. Finally, our monthly sampling frame limited our ability to observe events occurring on a shorter time scale.

This study provides evidence that, in the setting of PCV, children remain the major route of pneumococcal entry into the household, a reservoir for maintaining carriage within households, and both the source and recipient of the majority of intra-household transmission. The 13-valent conjugate vaccine (Prevnar13, [Pfizer]) now in routine use in the United States contains capsular polysaccharide to serotype 6A, which will likely confer cross-protection to serotype 6C [37], [38], and 19A; 19A and 6C are the two most frequently acquired and transmitted serotypes in this cohort. The CDC currently recommends a single PCV13 catch-up dose for children ages 2–5 years previously vaccinated with PCV7 [39]. Given the important role of older children in household pneumococcal dynamics, this catch-up schedule likely accelerates PCV indirect effects by limiting household introduction and transmission of vaccine serotypes. Finally, while our results provide insight into pneumococcal transmission in a high-burden population, transmission dynamics may differ in other settings according to risk factor distribution and prevalence of carriage. Longitudinal household studies will continue to be an important tool, defining pneumococcal carriage dynamics as new conjugate vaccines are introduced around the world.

## Supporting Information

Figure S1 **Seasonal trends in individual acquisition (⧫) and household introduction (•).** Incidence rate ratios and 95% confidence intervals obtained from Poisson regression analysis (with January as reference month of study entry).
(TIF)
