# Correction: Nasopharyngeal Carriage and Transmission of *Streptococcus pneumoniae* in American Indian Households after a Decade of Pneumococcal Conjugate Vaccine Use

**DOI:** 10.1371/journal.pone.0093878

**Published:** 2014-03-26

**Authors:** The PLOS ONE Staff

There is an error in Table 1. All values in the IQR column under “Household Characteristics” are preceded by “??”. A correct version of the table can be found below.

**Table 1:** : Characteristics of individuals and households enrolled in the study

Household characteristics (n = 300)	Median	IQR						
# of members (by interview)	6	[5, 7]						
# of reported members not enrolled in study	2	[1, 3]						
# of members under age 6 (by interview)	2	[1, 3]						
# of reported members under age 6	0	[0, 1]						
not enrolled in study								
# rooms in house	6	[4, 7]						
# sleeping rooms in house	3	[2, 3]						

Household risk factors	Proportion of households	%						
Running water	250/299	83.6%						
Toilet	245/299	81.9%						
Smoker in house	78/297	26.3%						
Wood-burning stove	184/298	61.7%						

Individual characteristics: children (#, % of enrollees in age category)	<2 years		2-4 years		5-8 years^b^		All children <9 years	
	#	%	#	%	#	%	#	%
Male	118	50.9%	114	51.4%	89	52.7%	321	51.5%
Breastfed during study	72	31.0%	19	8.2%	4	2.4%	95	15.2%
Daycare during study^a^	10	4.3%	16	6.9%	3	1.8%	29	4.7%
Otitis media during study	69	29.7%	26	11.2%	12	7.7%	108	17.3%
Antibiotics during study	75	32.3%	38	16.4%	19	11.2%	132	21.2%
PCV7 (3 doses)	192	82.8%	206	92.8%	121	72.0%	520	83.5%
Total in age category (% of all participants)	232	21.6%	222	20.7%	169	15.6%	623	58.1%

Individual characteristics: adults (#, % of enrollees in age category)	17- 39 years^c^		40-64 years		≥65 years		All adults ≥17 years	
	#	%	#	%	#	%	#	%
Male	84	22.2%	13	21.1%	2	22.2%	99	22.0%
Antibiotics during study	49	12.9%	10	16.4%	1	9.1%	60	13.4%
Smoked during study	56	14.8%	5	1.3%	0	0.0%	61	13.6%
Chewed tobacco during study	24	6.4%	3	0.8%	1	0.3%	28	7.4%
PS23 (any previous administration)^d^	18	4.8%	17	27.9%	8	72.7%	43	9.6%
Total in age category (% of all participants)	379	35.3%	61	5.7%	9	0.8%	449	42.1%

a Daycare attendance was defined as ≥4 hours per week outside the home with 2 or more children from other households

b Includes one child 10 years of age

c Includes two parents <17 years

d An additional 4 children <9 years of age reported receipt of PS23
