# Supplementary figures and images for: Nasopharyngeal Carriage and Transmission of Streptococcus pneumoniae in American Indian Households after a Decade of Pneumococcal Conjugate Vaccine Use
Source: PLoS One. 2014 Jan 17;9(1):e79578. doi: 10.1371/journal.pone.0079578 (PMC3894936; doi:10.1371/journal.pone.0079578)

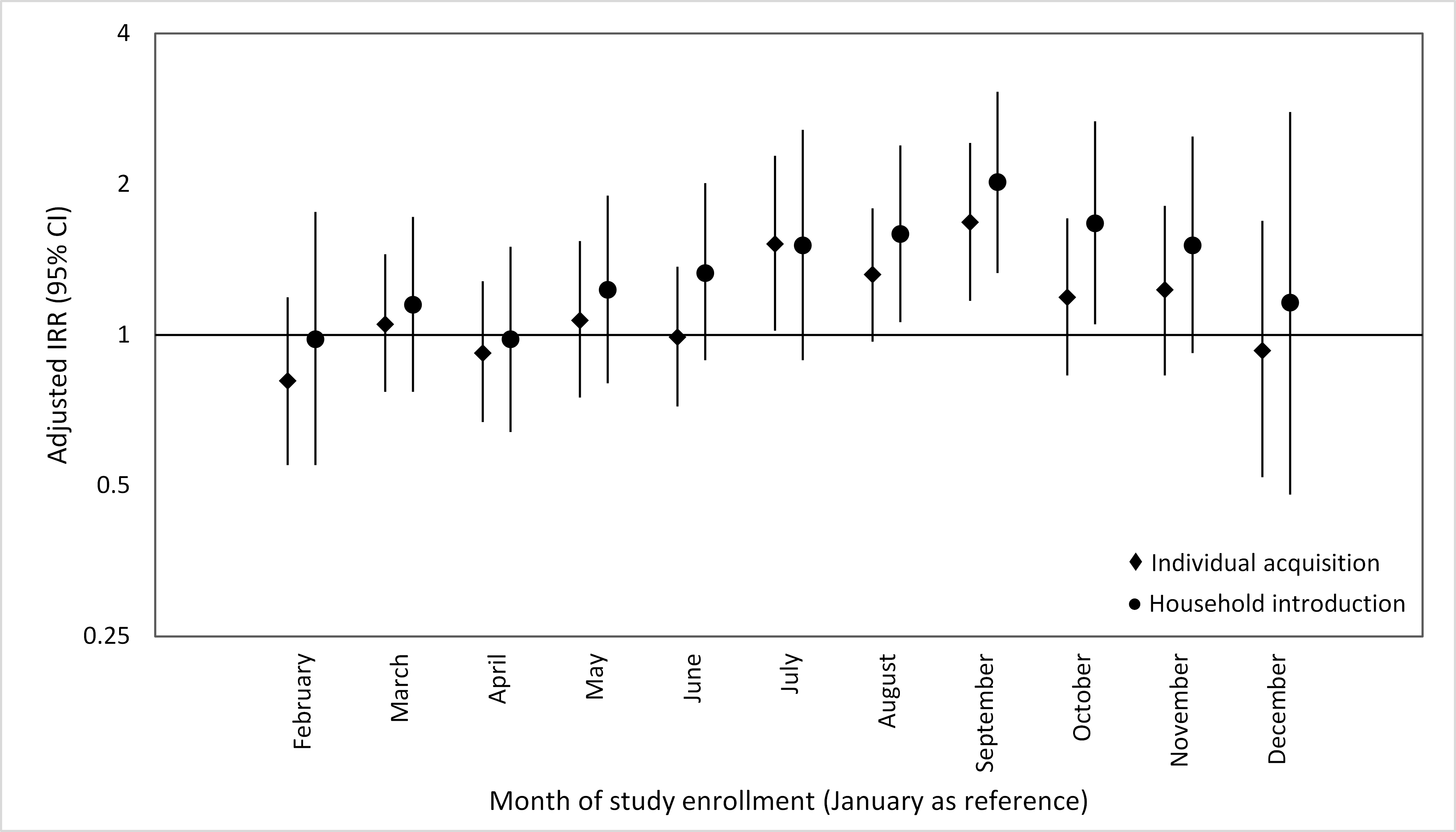

Supplement: Figure S1 — Seasonal trends in individual acquisition (⧫) and household introduction (•). Incidence rate ratios and 95% confidence intervals obtained from Poisson regression analysis (with January as reference month of study entry). (TIF) [file pone.0079578.s001.tif]
